# Can Targeting Sphincter Spasm Reduce Post-Haemorrhoidectomy Pain? A Systematic Review and Meta-Analysis

**DOI:** 10.1007/s00268-022-06807-3

**Published:** 2022-11-10

**Authors:** James Jin, Hanson Unasa, Praharsh Bahl, Melbourne Mauiliu-Wallis, Darren Svirskis, Andrew Hill

**Affiliations:** 1 Department of Surgery, South Auckland Clinical Campus, The University of Auckland, Level 2, Esme Green Bldg, Middlemore Hospital, Private Bag 93311, Auckland, 1640 New Zealand; 2 Faculty of Medical and Health Sciences, School of Pharmacy, The University of Auckland, Auckland, New Zealand

## Abstract

**Background:**

Haemorrhoidectomy is often complicated by significant post-operative pain, to which spasm of the internal anal sphincter is thought to be a contributing factor. This study appraises the evidence behind interventions aimed at lowering sphincter spasm to relieve post-haemorrhoidectomy pain.

**Methods:**

A Preferred Reporting Items for Systematic Reviews and Meta-analyses compliant systematic review was conducted. Medline, EMBASE, and CENTRAL databases were systematically searched. All RCTs which compared interventions targeting the internal anal sphincter to relieve pain post excisional haemorrhoidectomy were included. The primary outcome measure was pain on the visual analogue scale.

**Results:**

Of the initial 10,221 search results, 39 articles were included in a qualitative synthesis, and 33 studies were included in a meta-analysis. Topical glyceryl trinitrate (GTN) reduced pain on day 7 (7 studies, 485 participants), with a mean difference and 95% confidence interval (MD, 95% CI) of −1.34 (−2.31; −0.37), *I*^2^ = 91%. Diltiazem reduced pain on day 3 on the VAS, and the MD was −2.75 (−398; −1.51) shown in five studies (*n* = 227). Botulinum toxin reduced pain on day 7, in four studies with 178 participants, MD −1.43 (−2.50; −0.35) *I*^2^ = 62%. The addition of Lateral Internal Sphincterotomy to haemorrhoidectomy reduced pain on day 2 in three studies with 275 participants, MD of −2.13 (−3.49; −0.77) *I*^2^ = 92%. The results were limited by high heterogeneity and risk of bias.

**Conclusion:**

Evidence suggests that lateral sphincterotomy, administration of botulinum toxin and the application of topical diltiazem or GTN can reduce post-operative pain after haemorrhoidectomy. Lateral sphincterotomy should not be routinely used due to the risk of incontinence.

**Supplementary Information:**

The online version contains supplementary material available at 10.1007/s00268-022-06807-3.

## Introduction

Haemorrhoids are common and can significantly affect quality of life [1]. Excisional haemorrhoidectomy remains the most effective procedure with the lowest recurrence for prolapsing haemorrhoids [2]. In the past 2 decades, considerable research aimed to improve post-operative pain after haemorrhoidectomy [3, 4]. After excisional haemorrhoidectomy, patients develop linear wounds that extend up to the anorectal ring at the site of the vascular pedicle [5]. The appearance of these wounds is similar to that of a fissure-in-ano, where hypertonia of the internal anal sphincter has been associated with anal fissure [6, 7]. It is believed that spasm of hypertonia of the internal sphincter is responsible for the discomfort after haemorrhoidectomy, especially during defecation. Mechanisms contributing to pain and hypertonia include incarceration of the smooth muscle fibres and mucosa at the transfixed vascular pedicle, and epithelial loss of the canal [2, 5].

Current research suggests the use of multimodal pain relief protocols after ambulatory anorectal surgery can reduce pain and opiate usage [4, 8]. Such protocols include opiate sparing protocols as well as adjunctive treatments [8]. In particular, specific to haemorrhoidectomy, targeting sphincter spasm has been proposed to reduce post-operative pain [9]. It is hypothesised that spasm of the internal anal sphincter contributes to significant pain after haemorrhoidectomy. Numerous interventions trialled have investigated this hypothesis from utilising anal stretch to lateral sphincterotomy and pharmacological treatments [10–12].

This systematic review and meta-analysis summarises and appraises evidence behind methods of pain relief focusing on the reduction of sphincter spasm as a mechanism of pain. The Grading of Recommendations Assessment Developments (GRADE) approach is used to appraise the certainty of evidence [13]. The results of this review can be used to develop clinical recommendations and provide future research direction.

## Methods

A systematic review and meta-analysis was conducted according the preferred reporting of systematic reviews and meta-analyses (PRISMA) guidelines [14]. The study was registered with PROSPERO-registration: CRD42021288125. The original registration was subsequently changed to restrict studies targeting sphincter spasm only.

### Data sources

A comprehensive literature search was carried out from inception until January 2022, using relevant medical subject headings and keywords. The search terms incorporated the following themes: 1. pain relief/sphincter spasm/analgesia, and 2. haemorrhoidectomy/haemorrhoids. Results were restricted to the English language. Medline, Embase and CENTRAL were searched to identify randomised clinical trials comparing techniques to reduce sphincter spasm to improve post-haemorrhoidectomy pain. The references of the identified trials were also searched to find additional trials for inclusion. Only full-texts available in English were selected for inclusion, and this did not impact on the number of final included texts. The full search strategy is documented in Online Appendix 1, and the protocol is outlined in Online Appendix 2.

### Eligibility criteria

#### Inclusion criteria

All RCTs that investigated treatments aimed at reducing pain by the mechanism of reducing sphincter spasm after haemorrhoidectomy were included. Examples included, but were not limited to topical medical therapy or procedural interventions or techniques such as botulinum toxin administration or sphincterotomy.

#### Exclusion criteria

Studies that did not compare an intervention aiming to reduce sphincter spasm were excluded. Studies that compared different techniques of haemorrhoidectomy were excluded, and studies that were non-randomised or did not involve haemorrhoidectomy were excluded.

#### Selection process

A multi-stage study selection process was conducted, starting with title and abstract screening followed by full-text review of eligible studies. Two independent reviewers (J.J and H.U) screened the search results against the inclusion and exclusion criteria at all stages. If there were disagreements, consensus was reached by discussion with the senior author.

#### Data collection process

Data were independently extracted according to a predefined form in Microsoft Excel (Redmond, Washington, USA) by two authors (H.U., P.B.), followed by validation of all extracted data by the first author. Data in a graphical format were transformed into a numerical format using WebPlotDigitizer v4.5 (Ankit Rohatgi, Pacifica, California, USA). Data were accessed from the published manuscript, and further data were not obtained from study investigators.

#### Data items

Study and participants characteristics were extracted according to a predefined form. The primary outcome of interest was pain measured on a 0–10 cm visual analogue scale (VAS) across the most frequently reported post-operative days. Time points with sufficient measurements taken were pooled in a meta-analysis. If pain was measured using a categorical method this was synthesised using a risk ratio as appropriate. The secondary outcomes included incidence of headache, incontinence, and measurements of wound healing.

### Meta-analysis

The results were synthesised using a meta-analysis if outcomes measured were deemed similar and combinable. Continuous outcomes were synthesised using mean difference (MD) along with 95% confidence intervals. If the mean and standard deviation (SD) for continuous outcomes were unavailable, these were estimated using median, range, or inter-quartile range using the methods of Wan and Luo et al. [15] Categorical outcomes were synthesised using Risk Ratio, with the risk of outcome in having the intervention versus without the intervention. Meta-analysis was conducted using the package ‘meta’ in R [16, 17]. A random effects meta-analysis was conducted as the default method, using the Mantel Haenszel method for pooling, with the results displayed as a forest plot. Continuity correction was given as 0.5 for all zero cell frequencies in categorical outcomes. Heterogeneity was measured using the I-squared statistic. Publication bias was evaluated using a funnel plot if there were ten or more studies. A sensitivity analysis was conducted on type of excisional procedure, based on open or closed haemorrhoidectomy.

### Risk of bias

The risk of bias was assessed by two study authors (J.J., P.B.) using the Cochrane Risk of bias 2.0 tool with the following outcome domains: Risk of bias from the randomisation process, deviations from intended interventions, missing outcome data, measurement of the outcome, and selection of the reported result [18].

### Certainty of evidence

The certainty of evidence was assessed using the Grading of Recommendations Assessment Development and Evaluation (GRADE) approach [13]. Factors assessed include the risk of bias, precision of effect estimates, consistency of individual study results, directness of evidence, and publication or reporting biases. The GRADEpro software was used to deliver the final certainty of evidence rating [19]. Interpretation of GRADE certainty of evidence is outlined in Online Appendix 1.

## Results

**Fig. 1:**
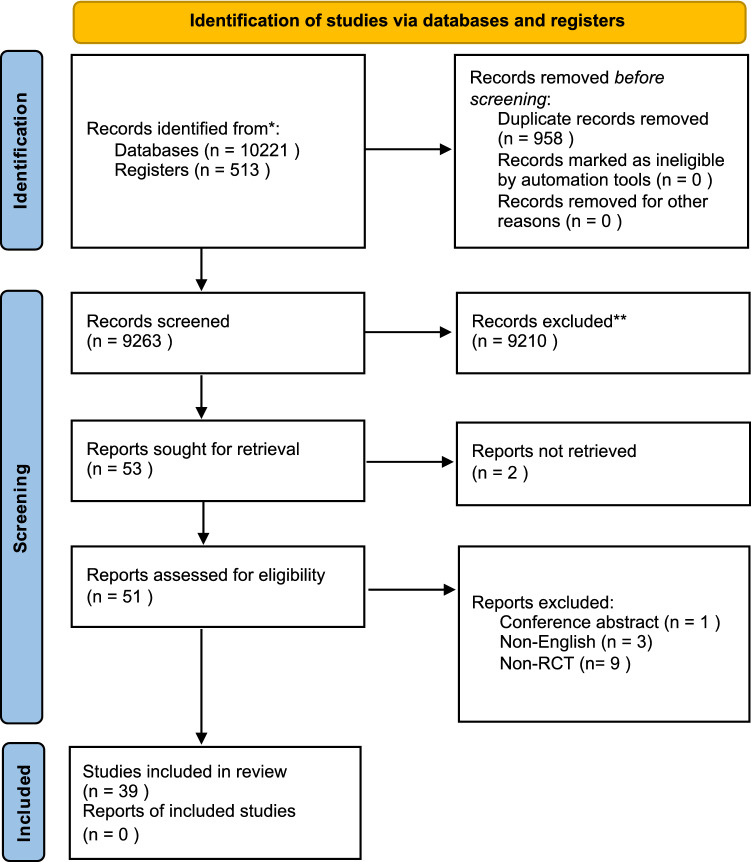
PRISMA diagram of included studies

**Fig. 2:**
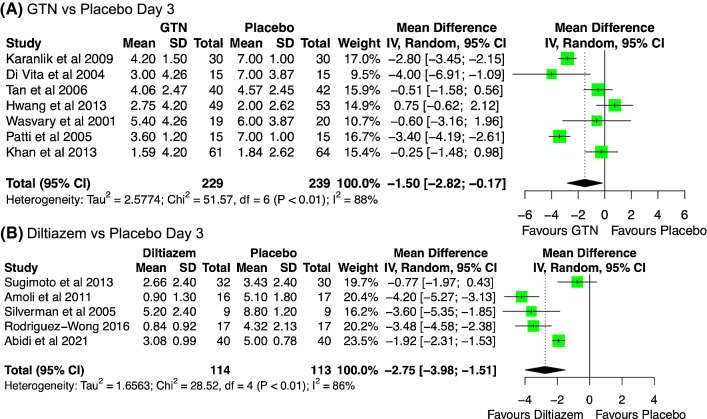
Forest plot of pain on the VAS for (**A**) GTN vs placebo on day 3, and (**B**) Diltiazem vs placebo on day 3

Of 10,224 initial search results, 704 were reviewed as abstracts, and 51 full-texts were reviewed for consideration. Following review of full-texts, 12 articles were excluded, leaving 39 articles included in a qualitative synthesis. A total of 33 studies with combinable outcomes were included in a quantitative synthesis. The PRISMA flow diagram is displayed in Fig. 1. The characteristics of included studies are displayed in Table 1. Figure 2 shows the forest plots for pain measured on the VAS for Glyceryl Trinitrate (GTN) and diltiazem. The remainder of figures for each meta-analysis is supplied in Online Appendix 1.

**Table 1:** Characteristics of included studies

Study name	Year	Country	Journal	Total participants	Total per cent female	Mean age (SD)	Follow-up period (days)	Dose/duration/description of intervention	Haemorrhoidectomy technique	Prophylactic antibiotics on induction	Post-operative antibiotics	Adjunct pain relief	Local anaesthesia infiltration
*GTN versus Placebo*													
Asif	2020	Pakistan	Pakistan Journal of Medical and Health Sciences	110	38	35 (12)	14	0.2% GTN versus placebo for TDS 12 days	Open	n.r	n.r	n.r	n.r
Di Vita	2004	Italy	Annali Italiani di chirurgia	30	43	38 (19)	14	0.2% GTN TDS versus placebo	Open	n.r	Metronidazole 400 mg PO TDS for 1 week	Lactulose	n.r
Franceshilli	2013	Italy	International Journal of Colorectal disease	203	38	49 (11)	42	0.4% GTN TDS versus placebo for 6 weeks	Ligasure	1 g Cefazolin and 500 mg metronidazole	Not mentioned	Ketorolac and laxatives	0.75% Naropine 20mls
Hwang	2013	Korea	Diseases of the Colon & Rectum	102	64	42 (11)	21	0.2% GTN TDS versus placebo 14 days	Open with suture ligation	2 g Cefazolin	2 g Cefazolin for 4 days	Amfenac and laxatives	n.r
Karanlik	2009	Turkey	Diseases of the Colon & Rectum	60	48	36 (11)	42	0.2% GTN versus placebo	Closed with ligation	n.r	n.r	Paracetamol, naproxen, metamizole, laxatives	n.r
Khan	2013	Pakistan	International Journal of Surgery	125	49	43 (9)	42	0.2% GTN versus lidocaine versus 0.2% GTN + lidocaine TDS for 6 weeks	Open	n.r	n.r	Paracetamol, codeine sitz baths	n.r
Lashari	2020	Pakistan	Pakistan Journal of Medical and Health Sciences	124	34	36 (11)	21	0.2% GTN versus placebo TDS for 14 days	n.r	n.r	n.r	n.r	n.r
Mari	2013	Italy	Diseases of the Colon & Rectum	41	32	49 (9)	14	0.4% GTN ointment versus lidocaine gel for 14 days	Stapled Haemorrhoidopexy	n.r	n.r	Ketorolac and laxatives	n.r
Patti	2005	Italy	Chirurgia Italiana	30	43	35 (17)	14	0.2% GTN ointment	Open	n.r	Metronidazole 500 mg PO TDS 7 days	Diclofenac, laxatives	n.r
Soltany	2009	Iran	Surgery Journal Medwell Journals	44	100	35 (13)	28	0.2% GTN BD versus placebo	Open	n.r	n.r	Ibuprofen 400 mg TDS	n.r
Tan	2006	Singapore	British Journal of Surgery	82	26	43 (11)	42	0.2% GTN (Rectogesic) versus placebo	Open diathermy	n.r	n.r	Paracetamol, diclofenac, sitz baths	0.25% Bupivicaine
Vahabi	2019	Iran	The Surgery Journal	40	n.r	n.r	2	0.2% GTN versus placebo	Open	n.r	n.r	Laxatives	n.r
Wasvary	2001	USA	Diseases of the Colon & Rectum	39	49	52 (n.r.)	7	0.2% NTG ointment for 7 days versus placebo	Closed	n.r	n.r	Hydrocodone, paracetamol, NSAIDs, and laxative	No local anaesthesia
*DILTIAZEM versus Placebo*													
Abidi	2021	Pakistan	Journal Ayub Medical College	80	34	39 (8)	3	2% diltiazem gel TDS versus placebo	Open	n.r	Antibiotics	Oral analgesics, sitz baths	n.r
Amoli	2011	Iran	Colorectal Disease	33	18	53 (16)	7	2% diltiazem ointment versus Vaseline	Open with diathermy	n.r	Not mentioned	Paracetamol, codeine, pethidine and laxatives	n.r
Rodriguez-Wong	2019	Mexico	Revista de Gastroenterologia de Mexico	52	48	48 (n.r.)	42	2% diltiazem gel versus placebo	Closed with suture	n.r	Not mentioned	Ketorolac, laxatives and sitz baths	n.r
Rodriguez-Wong	2016	Mexico	Revista de Gastroenterologia de Mexico	34	44	46 (n.r.)	3	2% diltiazem gel TDS versus placebo	Closed	n.r	Not mentioned	Ketorolac and sitz baths	n.r
Silverman	2005	USA	Diseases of the Colon & Rectum	18	61	45 (11)	7	2% diltiazem versus Vaseline TDS	Closed	n.r	n.r	Hydrocodone and laxatives	n.r
Suchdev	2014	Pakistan	Pakistan Journal of Surgery	80	18	39 (15)	3	2% diltiazem TDS versus placebo	Open	n.r	Metronidazole 400 mg TDS 3 days	Diclofenac, stool softener and sitz baths	n.r
Sugimoto	2013	Japan	World Journal of Surgery	62	58	63 (14)	14	2% diltiazem gel TDS versus methylcellulose gel	Harmonic Scalpel, Closed with suture	n.r	n.r	Loxoprofen 60 mg TDS	n.r
*Botulinum Toxin versus Placebo*													
Alvandipour et al	2021	Iran	Journal of Research in Medical Sciences	67	36	n.r	14	Botox 40 IU intersphincteric bilateral	Open	None	None	Intravenous apotel and morphine PRN	n.r
Davies	2003	England	Diseases of the Colon & Rectum	49	41	56 (12)	7	Botox 20 IU internal anal sphincter bilateral	Open with suture ligation	n.r	n.r	Codeine and paracetamol with laxatives	0.25% Bupivicaine 20mls
Patti	2005	Italy	Diseases of the Colon & Rectum	30	50	38 (16)	7	Botox 20 IU internal anal sphincter	Open-single suture fixation	Metronidazole on induction	Metronidazole 500 mg TDS 1 week	Nimesulide 100 mg	n.r
Singh	2008	England	Colorectal Disease	32	25	53 (10)	14	Dysport 150 IU intersphincteric bilateral	Open with diathermy	n.r	Metronidazole 5 days	Paracetamol, NSAID, codeine	0.5% Bupivicaine 20 ml
Sirikurnpiboon	2020	Thailand	Journal of the Anus, Rectum and Colon	82	46	42 (13)	180	Botox 30 IU intersphincteric space	Closed	n.r	Metronidazole for 1 week	Paracetamol, diclofenac, norfloxacin	n.r
*Lateral internal sphincterotomy versus none*													
Butt	2018	Pakistan	Pakistan Journal of Medical and Health Sciences	60	33	46 (8)	14	Sphincterotomy to left side open wound 1 cm to the dentate line	Open	Ceftriaxone 1 g metronidazole 500 mg × 3 doses	n.r	n.r	n.r
Chauhan	2009	India	Journal of Postgraduate Medicine	102	31	48 (9)	14	Sphincterotomy to dentate line	Open	n.r	n.r	Tramadol and sitz baths	n.r
Das	2013	Malaysia	International Journal of Collaborative Research on Internal Medicine & Public Health	50	24		90	Sphincterotomy through haemorrhoidectomy wound up to 1 cm upwards of dentate line	Open	2 g Cephalosporin and metronidazole × 3 doses	Total of 3 doses perioperatively	Laxatives and sitz baths	
Galizia	2000	Italy	European Journal of Surgery	42	48	41 (5)	n.r	Sphincterotomy up to dentate line	Open	n.r	n.r	n.r	n.r
Hosseini	2007	Iran	Archives of Iranian Medicine	120		44 (14)	14	Cutting one third of the internal sphincter by cauterisation	n.r	n.r	n.r	n.r	Under local anaesthesia
Kanellos	2005	Greece	World Journal of Surgery	78	50	51 (13)	7	LIS up to the dentate line	Open	n.r	n.r	n.r	n.r
Khubchandani	2002	USA	Diseases of the Colon & Rectum	42	48	52 (11)	n.r	Sphincterotomy not further specified	Closed	n.r	n.r	Paracetamol and oxycodone	n.r
Lu	2013	China	World Journal of Gastroenterology	192	49	48 (n.r.)	n.r	Lower edge of the internal sphincter was divided and cut off at the interscalene in the 3 or 9 o’clock position	Open	2 g Cephalosporin	n.r	n.r	n.r
Mathai	1996	Singapore	British Journal of Surgery	33	45	40 (9)	180	Internal sphincterotomy in haemorrhoidectomy wound up to dentate line	Open	n.r	n.r	Ketoprofen and laxatives	n.r
Sushel	2015	Pakistan	J Liaquat Uni Med Health Sci	116	34	42 (10)	10	Internal sphincter divided up to the dentate line through one of the haemorrhoidectomy wounds	Open	n.r	n.r	Sitz baths	n.r
Raza	2013	Pakistan	Journal of Rawalpindi Medical College	108	36	43 (n.r.)	28	Sphincterotomy not further specified	Open	n.r	n.r	n.r	n.r
Vijayaraghavalu	2021	India	Cureus	200	40	42 (11)	720	1 cm lateral incision 3 o’clock	Open with suture fixation	n.r	Metronidazole for 5 days	Topical magnesium sulphate, sitz baths	n.r
*Other*													
Ho	1998	Singapore	British Journal of Surgery	160	53	50 (12)	70	trimebutine 120 mg and Ruscogenins 10 mg (Proctolog) versus no intervention	Open diathermy	n.r	n.r	Ketoprofen, laxatives, sitz baths	n.r
Perrotti	2010	Italy	Canadian Journal of Surgery	270	42	49 (13)	14	0.3% nifedipine + 1.5% lidocaine ointment versus 1.5% lidocaine ointment	Open	n.r	n.r	n.r	n.r

*n.r.* not reported, *GTN* glyceryl trinitrate, *NTG* nitroglycerin, *LIS* lateral internal sphincterotomy

### Topical glyceryl trinitrate (GTN)

Thirteen RCTs compared GTN against placebo [20–32]. Intervention groups were randomised to the application of topical GTN (0.2–0.4%) applied peri-anally to the haemorrhoidectomy wound two to three times daily.

#### Post-operative day 1

Nine studies with 549 participants reported pain measured on day 1 post-operation using the 0–10 VAS. Topical GTN significantly reduced post-operative pain compared to placebo. [MD: −1.03, 95% CI (−2.03, −0.023), *p* = 0.045]. Heterogeneity was significant, *I*^2^ = 91%. (Fig. 1, Online Appendix 1).

#### Post-operative day 2

Seven studies reported results on post-operative day 2. Topical GTN significantly reduced pain compared to placebo [MD: −2.06, 95% CI (−3.44; −0.67), *p* = 0.036], with significant heterogeneity, *I*^2^ = 90%. (Fig. 2, Online Appendix 1).

#### Post-operative day 3

**Fig. 3:**
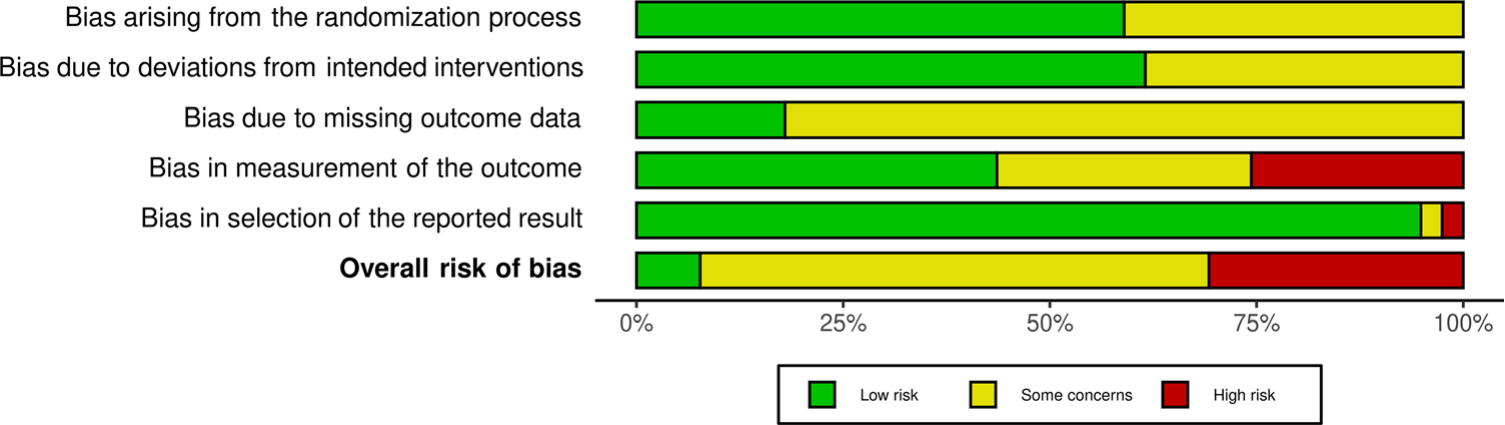
Risk of bias summary chart according to ROB 2.0

Similarly for post-operation day 3, a significant difference was observed across seven studies and 468 participants [MD: −1.50, 95% CI (−2.82; −0.17), *p* = 0.027]. There was significant heterogeneity *I*^2^ = 88%. (Fig. 3, Online Appendix 1).

#### Post-operative day 7

Seven studies with 485 participants showed that pain was significantly reduced on day 7 post-operation, [MD: −1.34, 95% CI (−2.31; −0.37), *p* = 0.012] *I*^2^ = 91%. (Fig. 4, Online Appendix 1).

#### Wound healing at 3 weeks

Wound healing at 3 weeks was reported in three studies with 252 participants [23, 24, 30]. The relative risk (RR) was 1.73, 95% CI (1.26; 2.36) *p* = 0.01, *I*^2^ = 0%, suggesting that participants were 73% more likely to have wounds fully epithelialized at 3 weeks. Among the studies, a total of 47% of participants had fully epithelialized wounds at 3 weeks. (Fig. 5, Online Appendix 1).

#### Incidence of headache

Headache was reported in 10 studies and results of nine studies were synthesised in a meta-analysis involving 679 participants. A random effects model indicated GTN use was associated with a sixfold increase in the incidence of headache [RR = 6.11, 95% CI (2.11–17.70) *p* = 0.004], with low heterogeneity *I*^2^ = 15%. (Fig. 6, Online Appendix 1).

#### Analgesic use

Two studies did not report pain on standardised scale, but rather reported analgesic use [26, 29]. Analgesic use was not able to be compared between studies, as studies reported different types and modes of analgesia given. Only one study reported pain on defecation [21]. Among trials involving GTN, there were no studies of anal manometry.

### Diltiazem

Seven studies evaluated the effect of topical diltiazem against placebo [33–39]. Intervention groups were randomised to the application of 2% topical diltiazem to the haemorrhoidectomy wound two to three times daily.

#### Post-operative day 1

Five studies with 227 participants show pain on the VAS was significantly reduced on post-operation day 1, [MD: −2.41, 95% CI (−3.71; −1.09) *p* < 0.001] with high heterogeneity, *I*^2^ = 84%. (Fig. 7, Online Appendix 1).

#### Post-operative day 2

Similarly, for day 2, pain reduction was significant [MD: −2.94, 95% CI (−4.36; −1.51) *p* <0.001], *I*^2^ = 89%. This was analysed in four studies with 209 participants. (Fig. 8, Online Appendix 1).

#### Post-operative day 3

Pain on day 3 was also significantly reduced on the VAS, [MD: −2.75, 95% CI (−3.98; −1.51) *p* < 0.001], *I*^2^ = 86%, shown in five studies 227 participants. (Fig. 9, Online Appendix 1).

#### Post-operative day 7

Pain on day 7 was measured in three studies and 113 participants [MD: −2.01, 95% CI (−3.92; −0.1) *p* = 0.038, *I*^2^ = 93%]. (Fig. 10, Online Appendix 1).

#### Other outcomes

The primary outcome was pain on the VAS for six of the seven studies. One study reported the primary outcome as time to complete wound healing, defined by complete epithelisation of the wound [36]. One study presented results of pain on defecation [39]. Analgesia amount was presented by five studies, however the method of measuring analgesia taken were heterogenous, thus was unable to be synthesised [33–35, 37, 39]. Anal manometry was not reported in any study comparing diltiazem.

### Botulinum toxin

Five studies compared the administration of botulinum toxin against placebo [40–44]. Participants in the intervention groups had botulinum toxin injected at the site of the internal anal sphincter intraoperatively.

#### Post-operative day 1

Pain measured with the VAS on day 1 in five studies with 260 participants show pain was significantly reduced, [MD: −.47, 95% CI (−2.04; −0.91) *p* <0.001], with low heterogeneity, *I*^2^ = 0%. (Fig. 11, Online Appendix 1).

#### Post-operative day 2

Pain on day 2 from five studies with 227 participants show a significant reduction in pain, [MD: −2.75, (−3.98; −1.51) *p* < 0.001], with high heterogeneity, *I*^2^ = 86%. (Fig. 12, Online Appendix 1).

#### Post-operative day 7

Pain on day 7 was reported in four studies with 178 participants. A significant reduction was found with moderate heterogeneity [MD: −1.43, 95% CI (−2.50; −0.35), *p* = 0.009], *I*^2^ = 62%. (Fig. 13, Online Appendix 1).

#### Other outcomes

Anal manometry was assessed in two studies [42, 43]. Both studies reported the botulinum toxin group had significantly lower mean resting pressures compared to the control groups at week 6. Singh et al. reported there were no significant differences in mean resting pressures by week 12 [43]. Results of resting anal pressures are summarised in Table 1, Online Appendix 1.

Only two studies reported pain at day 14 [40, 43]. Pain on defecation was reported in three studies. Analgesia use was presented in two studies, Alvandipour and Sirikunbpoon.

### Lateral internal sphincterotomy (LIS)

A total of 12 studies compared routine haemorrhoidectomy with lateral internal sphincterotomy (LIS) against haemorrhoidectomy without LIS [45–56].

#### Post-operative day 2

Pain was analysed at day 2 in three studies with 275 participants. The addition of LIS to haemorrhoidectomy reduced pain on the VAS, [MD: −2.13, 95% CI (−3.49; −0.77), *p* = 0.0021]. Heterogeneity was significant at *I*^2^ = 92%. Insufficient information was available to analyse pain on the VAS at other time points. (Fig. 14, Online Appendix 1).

#### Other measurements of pain

Pain was measured categorically in five studies [49, 50, 52, 54]. The severity of pain was categorised as mild pain, moderate pain and severe pain, with some studies defining ‘severe pain’ as pain > 6 on the 1–10 scoring system. Five studies with 614 patients show patients who had LIS in addition to haemorrhoidectomy were half as likely to have ‘severe pain’ at day 7–10 post-operation [RR: 0.47, 95% CI (0.32; 0.69) *p* = 0.009]. There was no significant heterogeneity *I*^2^ = 0%. (Fig. 15, Online Appendix 1).

#### Incontinence

Nine studies reported incontinence, defined as any incidence of new gas, liquid or solid faecal incontinence measured at a median follow-up of 4 weeks. Those who underwent lateral sphincterotomy were more than twice as likely to have any symptom of faecal or flatus incontinence in the early post-operative period [RR: 2.26, 95% CI (1.42; 3.58) *p* = 0.0035]. (Fig. 16, Online Appendix 1).

#### Other outcomes

Anal manometry was measured in three studies, of which two reported a significant decrease in mean resting pressures in the LIS group [48, 49]. The summary of anal manometry results are reported in Table 1, Online Appendix 1. Two studies reported wound healing at different time points.

### Sensitivity analysis

Sensitivity analysis based on type of haemorrhoid excision resulted in a reduction in number of studies when comparing open and closed haemorrhoidectomy separately. This resulted in a widened confidence interval for outcome analyses. Diltiazem versus placebo for open haemorrhoidectomy showed a larger effect in favour of diltiazem, compared to closed haemorrhoidectomy; however, this was based on two studies. The results of the sensitivity analysis are displayed in Table 2, Online Appendix 1.

### Other interventions

Ho et al. study investigated the effect of the “Proctolog” (trimebutine 120 mg and Ruscogenins 10 mg) suppository to reduce anal spasm, and found resting pressures were reduced, however had no effect on pain [57]. Perrotti et al. investigated the addition of nifedipine to lidocaine after haemorrhoidectomy, which did not show a conclusive improvement in pain [58].

### Risk of bias

The risk of bias was assessed using ROB 2.0. The majority of studies had ‘some concerns’ and 30.7% of studies had a ‘high’ risk of bias. The main source of bias related to the measurement of the outcome, where outcome assessors were aware of the intervention, and their knowledge of it may have influenced the outcome. Another source of bias arises from domain 3, bias due to missing outcome data. Although the majority of studies analysed results in a modified intention to treat analysis, 79.5% of studies did not relay any information regarding missing outcome data. The risk of bias summary is shown in Fig. 3. Risk of bias for individual studies is displayed in Fig. 17, Online Appendix 1.

### GRADE certainty of evidence

The GRADE certainty evidence for the reduction in pain for was generally ‘low’ for pain outcomes for all interventions, due to serious bias and inconsistency. The certainty of evidence for adverse event outcomes of headache and incontinence was high, due to a large effect being observed. An overall summary of findings is displayed in Table 2, and the GRADE appraisal tables for each outcome is displayed in Figs. 18–21, Online Appendix 1.

**Table 2:** GRADE summary of findings table

Outcome	Number of RCTs	Number of Patients (Intervention vs Control)	Risk of Bias	Other considerations	Pooled effect estimate	Certainty
*GTN versus placebo*						
Day 1 (VAS)	9	270 versus 279	Serious	Very serious inconsistency	MD 1.03 cm lower (2.03 lower to 0.02 lower)	⊕◯◯◯ Very low
Day 3 (VAS)	7	229 versus 239	Serious	None	MD 1.5 cm lower (2.82 lower to 0.17 lower)	⊕⊕◯◯ Low
Day 7 (VAS)	7	243 versus 242	Serious	None	MD 1.34 cm lower (2.31 lower to 0.37 lower)	⊕⊕◯◯ Low
Wound healing	3	125 versus 127	Very Serious	None	247 more per 1000 (from 88 to 460 more)	⊕⊕◯◯ Low
Headache	10	334 versus 337	Not Serious	Very strong association	76 more per 1000 (from 16 to 248 more)	⊕⊕⊕⊕ High
*Diltiazem versus placebo*						
Day 1 (VAS)	5	112 versus 115	Serious	Serious inconsistency	MD 2.4 cm lower (3.71 lower to 1.1 lower)	⊕⊕◯◯ Low
Day 3 (VAS)	5	114 versus 113	Serious	Serious inconsistency	MD 2.75 cm lower (3.98 lower to 1.51 lower)	⊕⊕◯◯ Low
Day 7 (VAS)	3	57 versus 56	Serious	Serious inconsistency	MD 2.01 cm lower (3.92 lower to 0.11 lower)	⊕⊕◯◯ Low
*Botulinum toxin versus placebo*						
Day 1 (VAS)	5	127 versus 133	Serious	None	MD 1.27 cm lower (2.04 lower to 0.91 lower)	⊕⊕⊕◯ Moderate
Day 2 (VAS)	5	127 versus 133	Serious	Serious inconsistency	MD 1.98 cm lower (3.25 lower to 0.7 lower)	⊕⊕◯◯ Low
Day 7 (VAS)	4	88 versus 90	Serious	Serious inconsistency	MD 1.43 cm lower (2.5 lower to 0.35 lower)	⊕⊕◯◯ Low
*Lateral internal sphincterotomy versus none*						
Day 2 (VAS)	3	139 versus 136	Very serious	None	MD 2.13 cm lower (3.49 lower to 0.77 lower)	⊕◯◯◯ Very low
Incontinence	9	447 versus 441	Serious	Strong association	43 more per 1000 (from 14 to 88 more)	⊕⊕⊕⊕ High
Severe pain	5	249 versus 249	Very Serious	Strong association	170 fewer per 1000 (from 218 to 100 fewer)	⊕⊕⊕◯ Moderate

*GTN* glyceryl trinitrate, *VAS* visual analogue scale, *MD* mean difference

## Discussion

The results of this meta-analysis show a significant benefit in pain reduction across multiple time points after haemorrhoidectomy. Several measures are effective in reducing pain, and these include the topical treatments GTN and diltiazem, as well as procedures such as botulinum toxin injection and lateral internal sphincterotomy. The magnitude of reduction of pain levels on the VAS was clinically meaningful and statistically significant despite the studies having small sample size. The certainty of evidence is low when appraised using the GRADE approach due to a moderate or high risk of bias especially in the domain of outcome assessment. A low certainty of evidence indicates that confidence in the effect is limited and the true effect may be substantially different from the estimated effect.

This meta-analysis reported lateral internal sphincterotomy to be effective in reducing pain. Sphincterotomy remains the most effective treatment for chronic anal fissure; however, anal incontinence remains a risk as a recent meta-analysis showed incontinence rates estimated at 6% across all randomised controlled trials [6]. Alternative, safer options include botulinum toxin administration as well as topical therapies. Botulinum toxin has been shown to be an effective agent in both lowering sphincter pressures and reducing pain. It is administered as a single dose and it is safe and its effect is reversible; however, its routine use is limited by high cost.

Chemical sphincterotomy with topical agents is an alternative strategy, which avoids the irreversibility of a lateral sphincterotomy and the effects on incontinence. Both topical agents GTN and diltiazem are potent vasodilators and trigger smooth muscle relaxation [59]. Increased anal spasm following haemorrhoidectomy is thought to account for the increase in mean resting pressures (MRP) of the sphincter [7]. MRPs were only measured in 10% of included studies, all of which are studies involving botulinum toxin administration or lateral internal sphincterotomy. There were no studies which tested manometry on patients who were given topical therapy; therefore, the effect on sphincter spasm with topical agents remains unclear in the post-haemorrhoidectomy setting. An alternative mechanism of topical therapies is a hypothesised effect of increasing anodermal blood flow to improve healing. Only three studies report wound healing after GTN administration, which found a RR of 1.73 (1.26; 2.36) for wounds that had fully epithelialized at 3 weeks compared to placebo; however, these results are limited by measurement bias where study assesors were unblinded.

The results of this meta-analysis are limited by the high degree of heterogeneity. The variation in study methods and co-interventions may cause significant heterogeneity in the meta-analysis. The studies use different analgesic drugs perioperatively which may affect the pain score on day one post-operation. Furthermore, most studies did not discharge patients using a standardised pain management regime. The inconsistent use of adjunctive techniques across studies such as oral metronidazole may affect post-operative pain [60]. Pain levels are perceived differently depending on the population involved and also depending on how the scores are ascertained; whether scores were self-recorded by participant or ascertained by an assessor may contribute to bias and heterogeneity. Other limitations include the in-ability to pool analgesia data in a meta-analysis due to different types of analgesia used, and variation in frequency of measurement in VAS scores, which result in insufficient data for certain days to be analysed.

In conclusion, this meta-analysis reports a range of interventions targeting a reduction in sphincter spasm to be effective in reducing pain after haemorrhoidectomy. Procedural treatments such as sphincterotomy and botulinum toxin administration are effective for reducing pain; however, sphincterotomy carries a twofold risk of early incontinence compared to haemorrhoidectomy alone. Botulinum toxin is an effective option; however, its routine use may not be cost-effective for pain relief alone. Pharmacologic reduction of sphincter tone could be a viable option, as it is safe and effective with few drawbacks. GTN is effective; however, there is a risk of headache. Diltiazem could be a promising treatment option, due to the absence of adverse effects. More well-designed randomised trials are required to investigate the effect of diltiazem post-haemorrhoidectomy.

## Supplementary Information

Below is the link to the electronic supplementary material.

Supplementary file1 (DOCX 4728 kb)

Supplementary file2 (DOC 85 kb)
