# Supplementary material for: Can Targeting Sphincter Spasm Reduce Post-Haemorrhoidectomy Pain? A Systematic Review and Meta-Analysis
Source: World J Surg. 2022 Nov 10;47(2):520–33. doi: 10.1007/s00268-022-06807-3 (PMC9803754; doi:10.1007/s00268-022-06807-3)
Supplement: Supplementary file 1 — Supplementary file1 (DOCX 4728 kb) [file 268_2022_6807_MOESM1_ESM.docx]

**Appendix 1.**

Search strategy Medline and Embase:

Ovid MEDLINE(R) Epub Ahead of Print, In Process & Other Non-Indexed Citations, Ovid MEDLINE (R) Daily, and Ovid MEDLINE (R) 1946-Present

1 postoperative pain/ or pain intensity/ or pain/ or pain measurement/ or pain.mp. or pain assessment/ or pain severity/ 829449

2 analgesia/ or controlled study/ or analgesi*.mp. or analgesic agent/ 219642

3 anesthesia/ or anesthe*.mp. 428997

4 vas.mp. 62136

5 pain assessment/ or visual analog scale/ or controlled study/ or visual analog*.mp. 149507

6 vrs.mp. 1467

7 questionnaire/ or McGill Pain Questionnaire/ 627272

8 postoperative pain/ or infiltration.mp. 198982

9 opioid.mp. or opiate/ 129656

10 glyceryl trinitrate.mp. or glyceryl trinitrate/ 13339

11 laxative.mp. or laxative/ 4332

12 gtn.mp. 2293

13 diltiazem.mp. or diltiazem/ 9762

14 sphincterotomy/ or sphincterotomy.mp. 7935

15 calcium channel blocker.mp. or calcium channel blocking agent/ 7513

16 botulinum toxin A/ or botulinum toxin/ or botulinum.mp. or injection/ or botulinum toxin B/ 69298

17 botox.mp. or botulinum toxin A/ 11567

18 flavonoid/ or flavonoid.mp. 63334

19 antibiotic.mp. or antibiotic agent/ 259866

20 topical treatment/ or topical drug administration/ or topical.mp. 130653

21 haemorrhoid.mp. or hemorrhoid/ 5510

22 haemorrhoid*.mp. or hemorrhoidectomy/ 2423

23 hemorrhoid*.mp. or hemorrhoid/ 7178

24 piles.mp. 1722

25 21 or 22 or 23 or 24 9418

26 1 or 2 or 3 or 4 or 5 or 6 or 7 or 8 or 9 or 10 or 11 or 12 or 13 or 14 or 15 or 16 or 17 or 18 or 19 or 20 2501893

27 25 and 26 2830

1. **Meta-analysis results**

Figure 1. Pain on the Visual Analogue Scale (VAS) for GTN vs placebo day 1

Figure 2. Pain on the VAS for GTN vs placebo day 2

Figure 3. Pain on the VAS for GTN vs placebo day 3

Figure 4. Pain on the VAS for GTN vs placebo day 7

Figure 5. Wound healing at 3 weeks for GTN vs placebo

Figure 6. Incidence of headache for GTN vs placebo

Figure 7. Pain on the VAS for diltiazem vs placebo on day 1

Figure 8. Pain on the VAS for diltiazem vs placebo on day 2

Figure 9. Pain on the VAS for diltiazem vs placebo on day 3

Figure 10. Pain on the VAS for diltiazem vs placebo on day 7

Figure 11. Pain on the VAS for botulinum toxin vs placebo on day 1

Figure 12. Pain on the VAS for botulinum toxin vs placebo on day 2

Figure 13. Pain on the VAS for botulinum toxin vs placebo on day 7

Figure 14. Pain on the VAS for Haemorrhoidectomy with Lateral Internal Sphincterotomy (LIS) vs Haemorrhoidectomy alone on day 2

Figure 15. Categorical outcome of ‘severe pain’ for haemorrhoidectomy + LIS vs haemorrhoidectomy alone

Figure 16. Incidence of fecal or flatus incontinence after haemorrhoidectomy with lateral sphincterotomy vs haemorrhoidectomy alone

Table 1. Summary of anal manometry results

| Study | Manometry results |
| --- | --- |
| Patti et al 2005 (Botox vs placebo) | In the placebo group, the MRP was significantly raised (*P* < 0.05) on fifth postoperative day compared with the baseline preoperative values, whereas in the Tox group it was significantly reduced (*P* < 0.01; Fig. 1). In both groups, the MRP returned to preoperative levels 30 days after hemorrhoidectomy. |
| Singh et al 2008 (Botox vs placebo) | The MRP was significantly lower in the botulinum toxin group (mean 50.5 mmHg; 95% CI 39.77 - 61.23) compared with the placebo group (mean 64.94 mmHg; 95% CI 55.65 - 74.22) (P = 0.04) at week 6. At week 12 there was no significant difference in MRP between the two groups. |
| Galizia et al 2000 (LIS vs no LIS) | LIS was followed by a significant reduction in both resting and squeeze pressures; both sphincter length and the length of the high pressure zone were shortened, and sphincter function, as assessed by the rectoanal inhibitory reflex, was unaffected. |
| Hosseini et al 2007 (LIS vs no LIS) | MRP showed a significant reduction in patients who underwent internal sphincterotomy while there were no significant changes in MSP in both groups. |
| Mathai et al 1996 (LIS vs no LIS) | Mean resting and maximum anal squeeze pressures, studied 6 weeks and 3 months after operation, were generally lower in group 2, but were not significantly different. |

Table 2. Summary of sensitivity analyses with open and closed haemorrhoidectomy analysed separately.

| Sensitivity Analysis | | | | | |
| --- | --- | --- | --- | --- | --- |
| VAS outcomes | | | | | |
|  | Day 1 | Day 2 | Day 3 | Day 4 | Day 7 |
| GTN vs placebo open | 6 studies,  -0.84 (-1.97; 0.29) I2= 83% | 5 studies,  -2.37 (4.25; -0.48), I2=93% | 5 studies,  -1.34 (-3.08; 0.40), I2=91% | 2 studies,  -1,.83 (-2.60; -1.07), I2=0% | 3 studies,  -0.94 (-2.44; 0.56), I2=72% |
| GTN vs placebo closed | 2 studies,  -2.14 (-4.96; 0.67), I2= 85% | Insuf. studies | 2 studies,    -2.06 (-4.10; -0.03), I2= 63% | Insuf. studies | 2 studies,  -1.84 (-5.07; 1.38), I2= 92% |
| Diltiazem vs placebo open | 2 studies,  -1.98 (-2.62; -1.34), I2=16% | 2 studies,  -3.38 (-3.78; -2.98), I2=0% | 2 studies,  -3.00 (-5.24; -0.77), I2= 94% | Insuf. studies | Insuf. studies |
| Diltiazem vs placebo closed | 3 studies,  -2.53 (-4.87; -0.19), I2= 91% | 2 studies,  -2.28 (-5.24; 0.68), I2-95% | 3 studies,  -2.57 (-4.42; -0.73), I2= 84% | Insuf. studies | 2 studies,  -1.47 (-4.15; 1.20), I2=92% |
| Lateral Internal Sphincterotomy vs none | n.r. | No difference | n.r. | n.r. | n.r. |
| Other outcomes | | | | | |
| Sphincterotomy sevre pain | no change from primary analysis | | | | |
| Wound healing GTN | no change from primary analysis | | | | |

n.r= not reported, Insuf. Studies= Insufficient studies for analysis


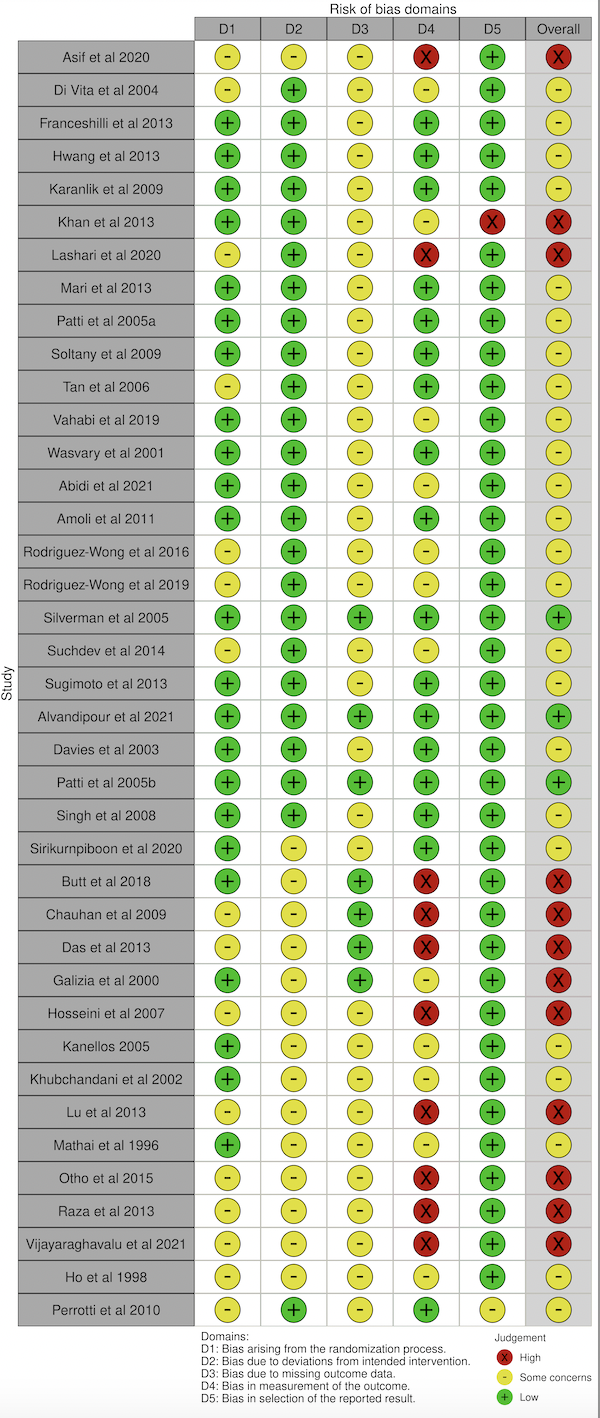


Figure 17. Risk of bias for individual studies according to Cochrane Risk of Bias 2.0.


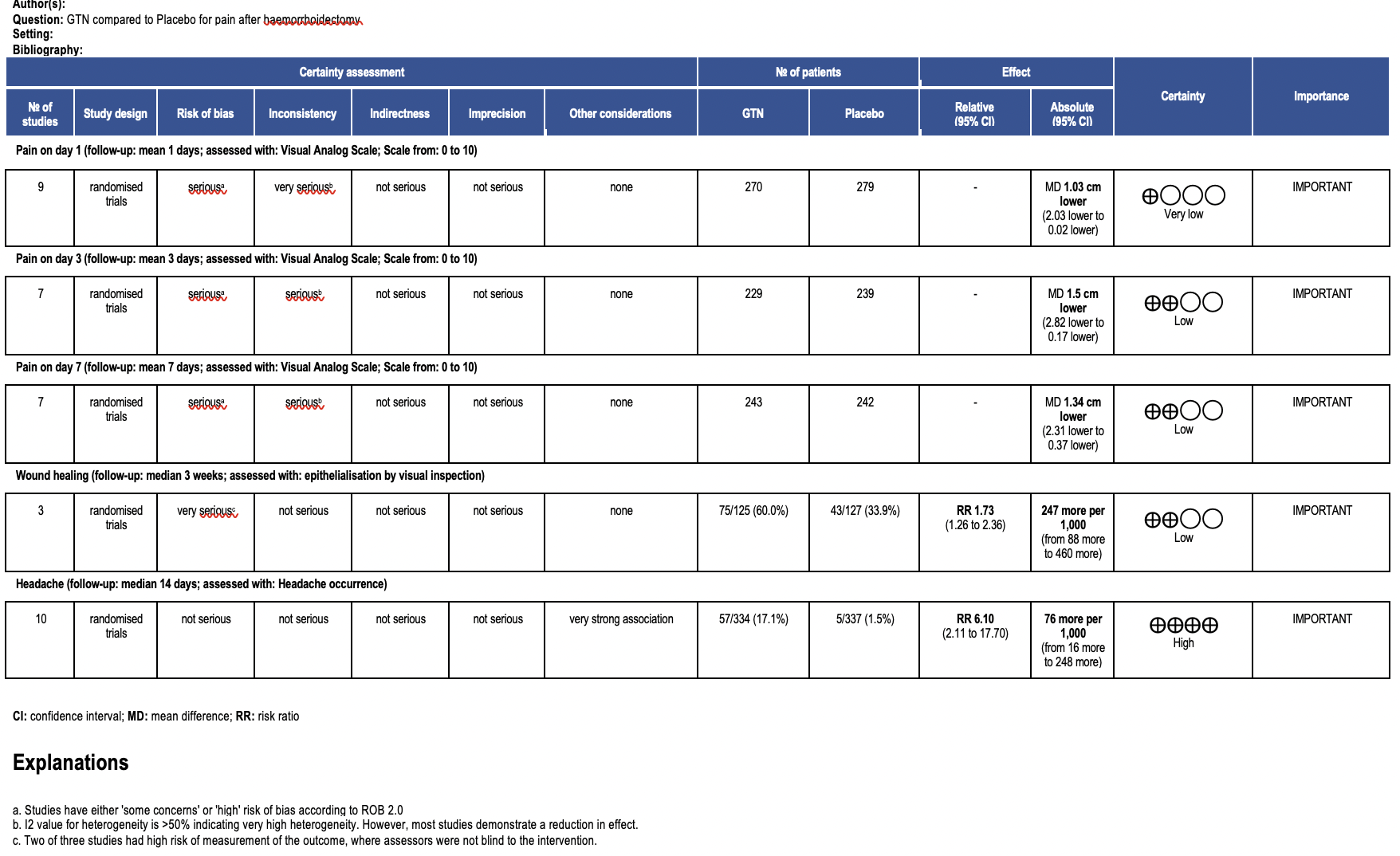


Figure 18. GRADE summary of findings for GTN vs placebo


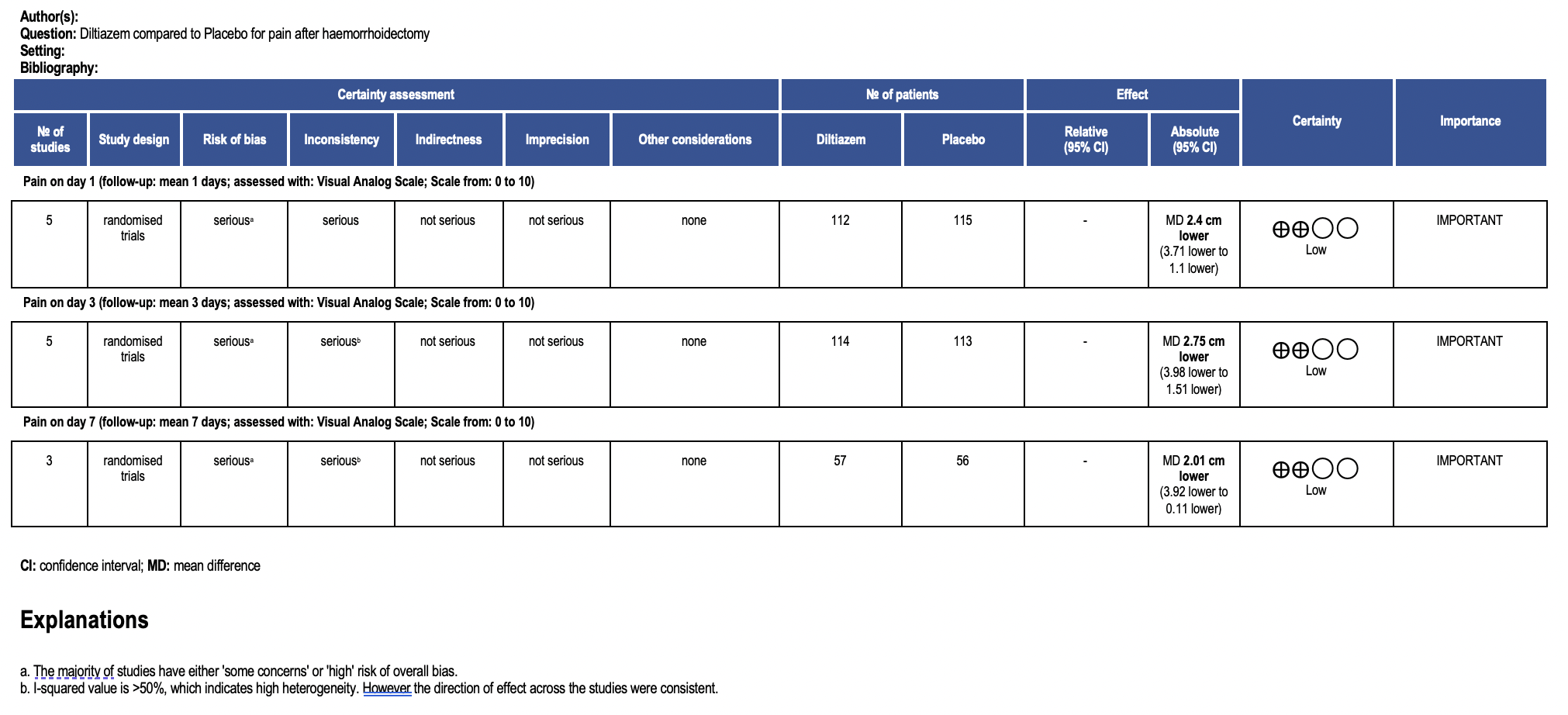


Figure 19. GRADE summary of findings for diltiazem vs placebo


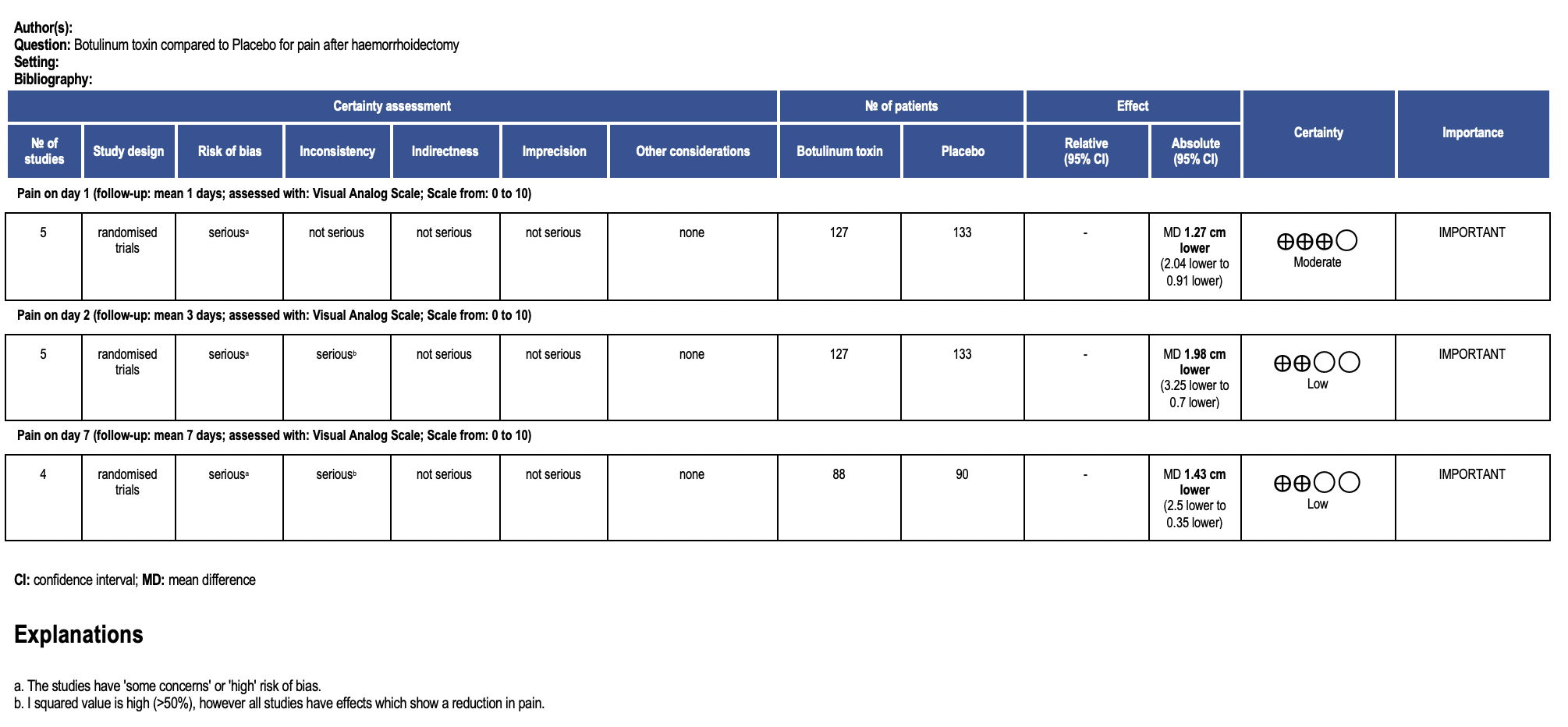


Figure 20. GRADE summary of findings for botulinum toxin vs placebo


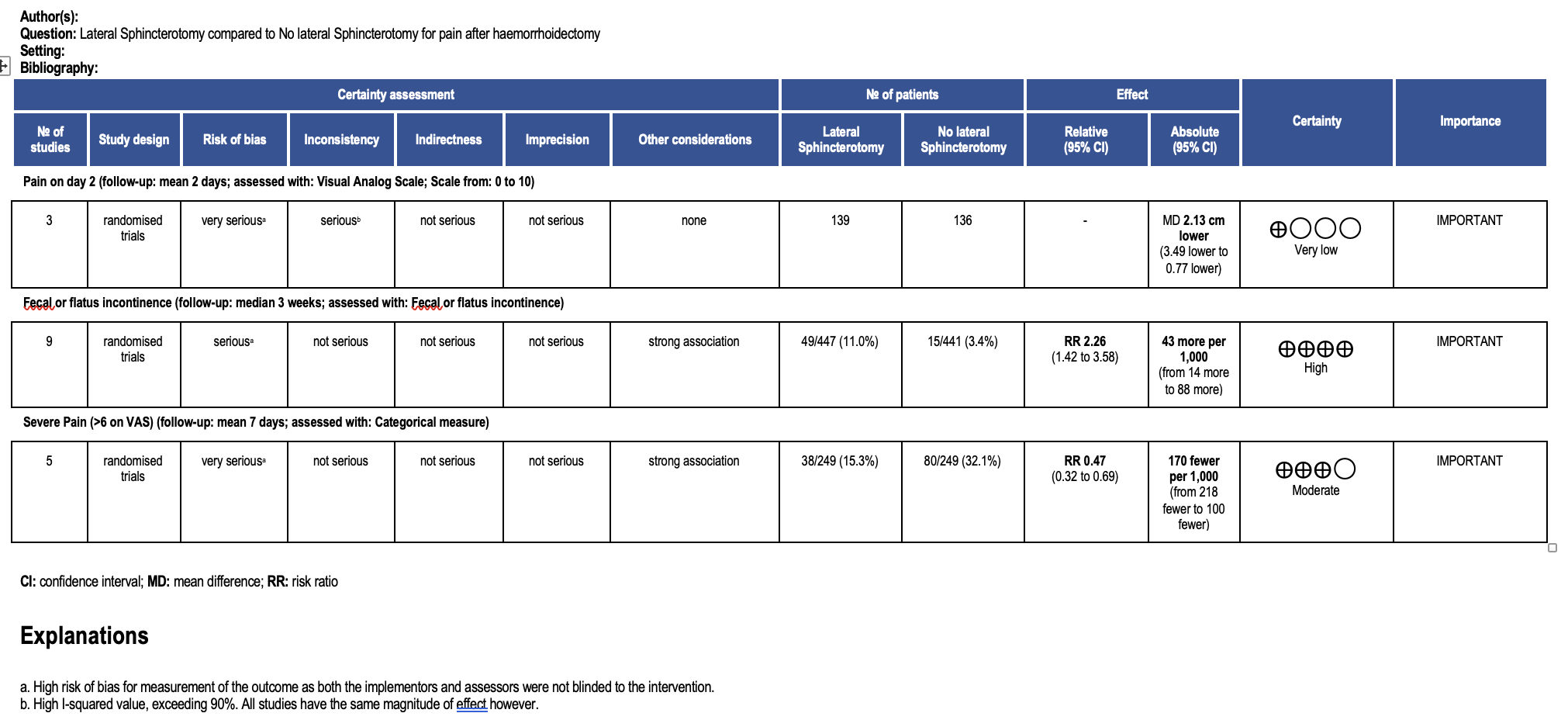


Figure 21. GRADE summary of findings for LIS vs no LIS after haemorrhoidectomy
