# Supplementary material for: Can Targeting Sphincter Spasm Reduce Post-Haemorrhoidectomy Pain? A Systematic Review and Meta-Analysis
Source: World J Surg. 2022 Nov 10;47(2):520–33. doi: 10.1007/s00268-022-06807-3 (PMC9803754; doi:10.1007/s00268-022-06807-3)
Supplement: Supplementary file 2 — Supplementary file2 (DOC 85 kb) [file 268_2022_6807_MOESM2_ESM.doc]

**Systematic review protocol**

**Title: Can targeting sphincter spasm reduce pain after haemorrhoidectomy? A systematic review and meta-analysis**

**Protocol date: 31^st^ January 2022**

**A systematic review protocol according to PRISMA-P (Preferred Reporting Items for Systematic review and Meta-Analysis Protocols) 2015 checklist: recommended items to address in a systematic review protocol***

| Section and topic | Item No | Details |
| --- | --- | --- |
| ADMINISTRATIVE INFORMATION | | |
| Title: |  |  |
| Identification | 1a | This is a protocol of a systematic review. |
| Update | 1b | This systematic review protocol is not based on an update of a previous review. |
| Registration | 2 | PROSPERO CRD42021288125 |
| Authors: |  |  |
| Contact | 3a | Authors:  James Jin, BBiomedSc, MBChB^1^  Hanson Unasa, MBChB^1^  Praharsh Bahl, MBChB^1^  Melbourne Mauiliu-Wallis, MBChB, MPH^1^  Darren Svirskis, BHB, BPharm, PhD^2^  Andrew Hill, MBChB, MD, EdD, FRACS, FACS, FISS, FRSNZ^1^  Corresponding Author: Dr James Jin, South Auckland Clinical Campus, Level 2, Esme Green Bldg, Middlemore Hospital, Private bag 93311, Otahuhu 1640, New Zealand. |
| Contributions | 3b | Contributions:  J.J. Conceptualisation, study design, conducted search strategy, screening of search results, data extraction, data curation, data analysis, writing of manuscript, editing and review of manuscript  H.U. screening of search results, data extraction, editing and review of manuscript  P.B. data extraction, data analysis, editing and review of manuscript  M.M.W. study design, data curation, editing and review of manuscript  D.S. study design, editing and review of manuscript  A.H. Conceptualisation, study design, writing and review of manuscript |
| Amendments | 4 | No amendments made to the protocol |
| Support: |  |  |
| Sources | 5a | Dr Jin is supported by a Health Research of New Zealand Clinical Training Research Fellowship |
| Sponsor | 5b | None |
| Role of sponsor or funder | 5c | No role in study development |
| INTRODUCTION | | |
| Rationale | 6 | Haemorrhoidectomy is complicated by significant pain. Multimodal pain regimens are effective at reducing pain and opiate usage. One such technique is reducing sphincter spasm. There are various techniques to reduce sphincter spasm after haemorrhoidectomy, and its effectiveness is not well elucidated in literature. Such interventions include post-operative administration of topical medical therapy, as well as surgical techniques such as anal stretch and sphincterotomy. |
| Objectives | 7 | This review aims to appraise the evidence of interventions targeting sphincter spasm in the reduction of pain after haemorrhoidectomy. Population: participants undergoing haemorrhoidectomy, Intervention: any intervention aimed at reducing sphincter spasm post-operatively, Comparator: control or placebo group. Outcome: Pain on the visual analogue scale |
| METHODS | | |
| Eligibility criteria | 8 | All randomised controlled trials are eligible for inclusion. Trials must have patients who have undergone haemorrhoidectomy and are randomised to an intervention which aims to target sphincter spasm to reduce pain as an outcome. All trials from 1980 onwards are considered. Language restriction to full texts in English. |
| Information sources | 9 | Search dates from September 2021 to December 2021. Medline, EMBASE, and CENTRAL databases will be searched. |
| Search strategy | 10 | Medline/Embase:  1 postoperative pain/ or pain intensity/ or pain/ or pain measurement/ or pain.mp. or pain assessment/ or pain severity/ 829449  2 analgesia/ or controlled study/ or analgesi*.mp. or analgesic agent/ 219642  3 anesthesia/ or anesthe*.mp. 428997  4 vas.mp. 62136  5 pain assessment/ or visual analog scale/ or controlled study/ or visual analog*.mp. 149507  6 vrs.mp. 1467  7 questionnaire/ or McGill Pain Questionnaire/ 627272  8 postoperative pain/ or infiltration.mp. 198982  9 opioid.mp. or opiate/ 129656  10 glyceryl trinitrate.mp. or glyceryl trinitrate/ 13339  11 laxative.mp. or laxative/ 4332  12 gtn.mp. 2293  13 diltiazem.mp. or diltiazem/ 9762  14 sphincterotomy/ or sphincterotomy.mp. 7935  15 calcium channel blocker.mp. or calcium channel blocking agent/ 7513  16 botulinum toxin A/ or botulinum toxin/ or botulinum.mp. or injection/ or botulinum toxin B/ 69298  17 botox.mp. or botulinum toxin A/ 11567  18 flavonoid/ or flavonoid.mp. 63334  19 antibiotic.mp. or antibiotic agent/ 259866  20 topical treatment/ or topical drug administration/ or topical.mp. 130653  21 haemorrhoid.mp. or hemorrhoid/ 5510  22 haemorrhoid*.mp. or hemorrhoidectomy/ 2423  23 hemorrhoid*.mp. or hemorrhoid/ 7178  24 piles.mp. 1722  25 21 or 22 or 23 or 24 9418  26 1 or 2 or 3 or 4 or 5 or 6 or 7 or 8 or 9 or 10 or 11 or 12 or 13 or 14 or 15 or 16 or 17 or 18 or 19 or 20 2501893  27 25 and 26 2830  CENTRAL:  Hemorrhoidectomy OR Hemorrhoid AND Clinical Trial OR RCT |
| Study records: |  |  |
| Data management | 11a | References are downloaded to Endnote (Clarivate analytics, London, UK) for organisation and screening. |
| Selection process | 11b | Two reviewers will independently screen titles and abstracts against the inclusion and exclusion criteria. |
| Data collection process | 11c | Data is extracted to a predefined form on Microsoft Excel (Microsoft, Redmond, Washington, USA) |
| Data items | 12 | Study author, year, journal published, intervention details, participant characteristics such as age, gender, grade of haemorrhoids, co-interventions, operative details, antibiotics on induction, or use of adjunctive techniques. Pain scores (continuous or categorical) across all time points measured, Pain on defecation, analgesia amount taken). |
| Outcomes and prioritization | 13 | The primary outcome is pain on the visual analogue scale (VAS), across all pa. Secondary outcomes include intervention specific adverse effects such as incontinence, headache etc., categorical outcomes of pain, amount of analgesia taken. |
| Risk of bias in individual studies | 14 | Risk of bias will be analysed at the study level using Cochrane risk of bias tool 2.0. |
| Data synthesis | 15a | A meta-analysis will be conducted for the primary outcome of pain on the VAS, if studies report pain outcomes using a continuous 10 point scale. Categorical outcomes are included in a meta-analysis if the population, interventions and outcomes are deemed similar. |
|  | 15b | Continuous data will be synthesised in a meta-analysis. Outcomes reported will be as a mean difference with 95% confidence intervals and p values. Categorical data is analysed as a risk ration with a confidence interval and p value. Heterogeneity is assessed using the I^2^. Statistic. |
|  | 15c | No sensitivity or subgroup analysis is planned. |
|  | 15d | Outcomes where quantitative synthesis is not feasible, ie if there were insufficient data, the results will be synthesised in a summary or a table. |
| Meta-bias(es) | 16 | Publication bias will be assessed using a funnel plot if there are 10 or more studies in the synthesis. |
| Confidence in cumulative evidence | 17 | The certainty of evidence will be assessed using the GRADE approach. |
